# Is the maturity of hospitals' quality improvement systems associated with measures of quality and patient safety?

**DOI:** 10.1186/1472-6963-11-344

**Published:** 2011-12-20

**Authors:** Oliver Groene, Nuria Mora, Andrew Thompson, Mercedes Saez, Mercè Casas, Rosa Suñol

**Affiliations:** 1 Avedis Donabedian University Institute, Autonomous University of Barcelona, Barcelona, Spain; 2 CIBER Epidemiology and Public Health (CIBERESP), Barcelona, Spain; 3 School of Social and Political Science, University of Edinburgh, UK; 4 IASIST, Barcelona, Spain; 5 Department of Health Services Research and Policy, London School of Hygiene & Tropical Medicine, London, UK

## Abstract

**Background:**

Previous research addressed the development of a classification scheme for quality improvement systems in European hospitals. In this study we explore associations between the 'maturity' of the hospitals' quality improvement system and clinical outcomes.

**Methods:**

The maturity classification scheme was developed based on survey results from 389 hospitals in eight European countries. We matched the hospitals from the Spanish sample (113 hospitals) with those hospitals participating in a nation-wide, voluntary hospital performance initiative. We then compared sample distributions and explored associations between the 'maturity' of the hospitals' quality improvement system and a range of composite outcomes measures, such as adjusted hospital-wide mortality, -readmission, -complication and -length of stay indices. Statistical analysis includes bivariate correlations for parametrically and non-parametrically distributed data, multiple robust regression models and bootstrapping techniques to obtain confidence-intervals for the correlation and regression estimates.

**Results:**

Overall, 43 hospitals were included. Compared to the original sample of 113, this sample was characterized by a higher representation of university hospitals. Maturity of the quality improvement system was similar, although the matched sample showed less variability. Analysis of associations between the quality improvement system and hospital-wide outcomes suggests significant correlations for the indicator adjusted hospital complications, borderline significance for adjusted hospital readmissions and non-significance for the adjusted hospital mortality and length of stay indicators. These results are confirmed by the bootstrap estimates of the robust regression model after adjusting for hospital characteristics.

**Conclusions:**

We assessed associations between hospitals' quality improvement systems and clinical outcomes. From this data it seems that having a more developed quality improvement system is associated with lower rates of adjusted hospital complications. A number of methodological and logistic hurdles remain to link hospital quality improvement systems to outcomes. Further research should aim at identifying the latent dimensions of quality improvement systems that predict quality and safety outcomes. Such research would add pertinent knowledge regarding the implementation of organizational strategies related with quality of care outcomes.

## Background

Since his landmark publication in 1966, numerous studies have addressed Avedis Donabedian's theory to understand health care quality in terms of structure, process and outcomes [1]. Initial debates focused on the validity of process versus outcome measures of quality. It is now commonly agreed that process measures should only be used if they have an established relationship with desired outcomes and in turn, outcomes measures should be used that can be linked to specific processes of care [2]. Substantial variations between hospitals with regard to both process and outcome indicators have been documented in numerous studies and persist in clinical practice [3-5].

More recently, calls have been made to bring back to the attention of the quality of care debate the 'forgotten dimension of structure', which includes, for example, the role of senior leadership, organizational management, incentive structures and information management [6]. While structures do not directly influence outcomes of care, they are important to shape the processes which are then indirectly and directly associated with quality of care outcomes. The structural dimension is addressed by most hospitals in developed countries, either as a statutory requirement or voluntarily, in terms of developing and implementing a range of strategies that are bundled under the overall hospitals' quality improvement systems. This may range from simple structural requirements (policies, mission statements, professional licensing requirements, and quality committees) to sophisticated measures such as data-driven systems that are deployed organization-wide.

While hospitals' investment in quality systems in terms of professional time, documentation systems and management are substantial, the evidence base of the impact of these systems at the level of clinical practice or patient safety is not well developed and research on this topic has only recently been gaining interest [7-11]. As part of the European project "Methods of Assessing Response to Quality Improvement Strategies (MARQuIS)" a classification model for hospital quality improvement systems was developed [12] which measured quality improvement, defined as 'the application of quality policies and procedures, quality governance structures, and quality activities to close the gap between current and expected levels of quality'. The model assesses 'maturity' in the sense of reflecting the developmental stage of various quality improvement strategies. It was developed based on internationally accepted evaluations of contributors to quality. Development and testing included grouping items into seven theoretically derived dimensions, using principal component analysis to assess loadings of items onto each factor and assessing internal consistency of each of the scales. The domain scores were combined in a mean overall score for each hospital. In order to further explore robustness of the maturity index three independent analyses were performed: hypothesis testing; on-site hospital visits; and expert assessment of the maturity of the QI system based on written information. Further details of the variables are explained in the methods section; details on the development and validation procedure are reported elsewhere [13]. Applying the maturity index to the hospitals participating in the MARQuIS study demonstrated that there is considerable variance in the development of the hospital quality systems within and between countries. Hospitals with higher developed quality improvement systems achieved better quality and safety outputs (such as providing patient ID bracelets or dispensers to facilitate hand hygiene) [14]. However, it has not been demonstrated yet whether hospitals with a more mature quality improvement system also perform better in terms of quality and patient safety outcome indicators, not just outputs. The objective of this paper is, therefore, to explore associations between the 'maturity' of the hospitals' quality improvement system (MI) and hospital-wide quality and patient safety outcomes. Given that the raison d'être of hospital quality improvement systems is to improve the quality of care, we hypothesize that hospitals with more mature quality systems perform better on these indicators.

## Methods

### Setting and participants

This cross-sectional study was performed in Spanish hospitals that participated in the MARQuIS study and at the same time contributed data to participate in a national, voluntary benchmarking initiative from IASIST, 20 Top Hospitals.

#### MARQuIS Project

An EU-funded research project to assess the effectiveness of quality improvement strategies in European hospitals in order to provide information on contracting requirements for patients moving across borders and for individual hospitals when reviewing the design of their quality strategies. The project ran from 2005 to 2009 and was led by the Avedis Donabedian University Institute, Autonomous University of Barcelona, Spain. Overall, 389 hospitals were recruited from eight countries (Belgium, Czech Republic, France, Ireland, the Netherlands, Poland, Spain, and the UK) to participate in the data collection. As part of the project a maturity classification model for hospital quality improvement systems was developed. The results of the project can be accessed free-of-charge in a supplement to Quality & Safety in Health Care: http://qshc.bmj.com/content/18/Suppl_1

#### IASIST 20 Top Hospitals

20 Top Hospitals is a voluntary, benchmarking initiative to which all Spanish hospitals can subscribe. In its tenth year since inception, this initiative provides information to top management on hospital level indicators and in six specific areas: nervous system, respiratory diseases, heart diseases, trauma, orthopedics and obstetrics. In 2009, 155 hospitals, representing about half of all Spanish hospitals in the National Health Service participate in this initiative. IASIST methodologies draw on a yearly database of more than 3 million discharges obtaining case-mix and risk adjusted clinical and non-clinical performance measures in order to inform clinical quality management and to provide top-management with information on hospital performance. More information can be found at: http://www.iasist.es/

The MARQuIS questionnaire was administered in 2006 by online-survey to 113 hospitals in Spain, 105 of which provided data to compute the maturity index on the development of the quality improvement system. Of these hospitals, 51 were also involved in the IASIST project in 2007 (reflecting 2006 hospital admissions). Permission was sought from hospitals to merge the databases from the MARQUIS and IASIST project in order to pursue the objective identified above. The final sample comprised 43 hospitals, where sufficient information and permission were provided for the study to take place.

### Measures

Outcome variables were obtained from the IASIST data and include quality and patient safety indicators at hospital level which are calculated on the basis of the administrative hospital discharge data set Minimum Basic Data Set (MBDS), a compulsory data set for all hospital admissions in Spain. We included four hospital-level indicators which are calculated based on the ratio of the observed to expected number of cases, taking into consideration hospital-specific case mix and severity (Table 1). Details on the indicators have been published by IASIST [15,16] and statistics and calibrations are attached in Additional File 1 Annex 1. All IASIST indicators for this analysis are based on 2007 data to match the data on the quality improvement system that was gathered within the MARQuIS project in the same year.

**Table 1:** Description of dependent variables

Indicator	Description	Risk adjustment
Adjusted hospital mortality index*	The number of deaths observed in the unit of analysis divided by the number of expected deaths.	Age, sex, risk of death for first diagnostic code, risk of death for second diagnostic code with maximum risk, risk of death for the procedure with maximum risk, type of admission (urgent/non-urgent), type of DRG (surgical/non-surgical), type of hospital (teaching/non- teaching hospital), hospital service contract (Public or Private), catchment area (urban/rural) and transfer policies of the hospital to long-term care.

Adjusted hospital complications index*, **	Complications that occur during the hospital stay divided by expected complications, including sentinel events and risk-adjusted complications.*	Age, risk of complications of the first diagnostic code, risk of complication of secondary code with maximum risk, risk of procedure with maximum risk, type of admission, type of DRG, type of hospital (teaching/non- teaching hospital), hospital service contract (Public or Private), and number of diagnostics for discharge.

Adjusted hospital readmissions index*	Readmission rates up to 30 days from first admission of a patient with readmission registered as urgent divided by expected readmissions.	Age, sex, type of admission, probability of readmission of the first diagnostic code of the initial admission, probability of readmission of the second diagnostic code with maximum risk of readmission, probability of readmission for procedure with maximum risk, average length of stay of initial admission, entity financing first admission, type of DRG, type of hospital (teaching/non- teaching hospital), hospital service contract (Public or Private).

Adjusted hospital length of stay index*	Sum of bed days consumed for each of the episodes in the unit of analysis divided by the sum of bed days expected for these episodes.	Age, sex, bed days expected for first diagnostic code, bed days expected for second diagnostic code, bed days expected for the procedure, type of admission, type of DRG, type of hospital (teaching/non- teaching hospital), catchment area (urban/rural), type of discharge hospital and hospital service contract (Public or Private),.

*values of the statistical models are reported in Additional File 2 Annex 1.

**see Additional File 1 Annex 1 for a full list of complications included.

For the main independent (predictor) variable, we used the MARQUIS Maturity Index (MI) of the hospitals' quality improvement system, which is based on a self-evaluation of the quality system by the hospital quality manager. The maturity index was developed as part of a larger study [12,13]. The QI maturity index consists of 113 items across seven domains: policy, planning & documentation (20 items); leadership (36 items); structure (19 items); general quality improvement activities (8 items); specific quality improvement activities (20 items); patient involvement (6 items); and accountability (4 items). The following gives an example for the type of items included in the maturity index:

*"Which of the following quality improvement activities take place in your hospital"*: [quality improvement teams or circles, internal audit, adverse event reporting and analysis, risk management and patient safety, patient surveys, analysis of patient complaints, monitoring the views of referring professionals, regular staff performance reviews]. Answers are scored on a four-point scale: *"1 = Yes, this activity takes places systematically in most departments (> 50%), 2 = Yes, this activity takes place in most departments (> 50%), but not systematically, 3 = Yes, this activity takes place in some departments (< 50%), 4 = No, this activity does not take place"*.

Responses to the items were weighted according to the plan-do-check-act (PDCA) cycle: items reflecting preparation or planning of a quality strategy received a lower weight than items reflecting that a strategy has been implemented, which in turn received a lower weight than an item reflecting that data on the implementation of a strategy was available and used to guide quality improvement efforts. Based on the mean scores of each of the dimensions, an aggregate score for the maturity of the hospitals' quality improvement system was computed. Previous research addressed the evaluation of the psychometric properties and validity in a larger sample of European hospitals and indicated good construct validity and concordance between self-assessment and expert review [13]. Moreover, research demonstrated that the MI can be used to distinguish hospitals with regard to the implementation of quality strategies and outputs [14,17]. In addition to the MI, various variables reflecting hospital structural characteristics, such as hospital ownership, type and size, were analyzed as potential predictor variables.

### Analysis

In a first step, we described measures of central tendency and dispersion for hospital characteristics and IASIST quality and patient safety indicators and compared hospital characteristics in the sample with those in the Spanish MARQuIS sample using Fisher's exact test and the Mann-Whitney *U*-test.

In a second step, we explored associations between dependent and independent variables. Distributions of all variables were tested using the Kolmogorov-Smirnov statistic. Analysis of correlations between the maturity index and the dependent variables were performed using appropriate parametric (Pearson's product moment correlation coefficient) and non-parametric methods (Spearman's correlation coefficient) [18]. Considering the limited sample size and the potential effect of outliers on the correlation coefficients, we performed the 'bootstrapping' technique in the R statistical software package [19]. In order to facilitate interpretation we report both original correlation coefficients and the related bootstrap results.

In a third and final step, multiple regression analysis was performed to assess the effect of MI after adjusting for hospital structural characteristics that might confound the associations. We used a multiple regression model with hierarchical variable entry to separately assess the effect of MI and the remaining structural characteristics of the hospital (such as hospital ownership, hospital type and size) on the dependent variables. We created dummy variables to introduce the categorical variables to the multiple regression models (university vs non-university hospitals; public vs private hospitals; and small vs medium vs large hospitals). In order to obtain normality or homocedasticity (constant variance) of dependent variables we used the Box-Cox transformation, estimating where appropriate the constant using the maximum verisimilitude function in the R statistical software [20]. Considering the existence of outliers in the dataset we compared the estimates of the ordinary least square regression with other, more robust methods of estimation [21]. Various models were assessed using either Huber weighting or bi-weighting and the model with the smallest standard error of residuals was selected. Similar to the correlation analysis, we then computed the bootstrap estimates and 95% confidence interval for the regression coefficients. Bootstrapping, Box-Cox transformations and robust regression were performed in R (version 2.10.1). The remainder of the analysis was performed using SPSS (v18).

## Results

**Figure 1:**
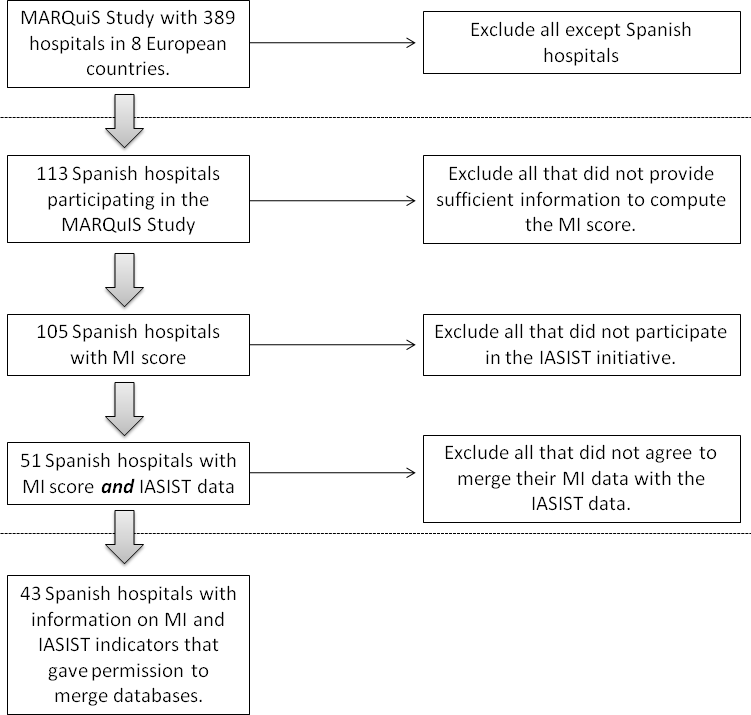
**Flowchart - Merging MARQuIS and IASIST data sets**.

Of the 113 hospitals participating in the Spanish MARQuIS and the 51 of these that participated in the IASIST project, 43 provided sufficient information on both data sets and agreed for the data sets to be merged (Figure 1). In Table 2 we provide an overview of the characteristics of these hospitals with regard to hospital type, ownership, size (in terms of number of beds) and characteristics of the maturity index variable of the hospitals' quality improvement system (MI).

**Table 2:** Distribution of hospitals in the sample

	Hospitals participating in the MARQuIS Project, SPAIN	Hospitals participating in MARQuIS, but not in IASIST	Hospitals participating in MARQuIS and IASIST	p-value
**N Hospitals**	**113**	**62**	**43**	

**Hospital type**				
**University**	32 (28.3%)	14 (23.0%)	16 (37.2%)	0.184^$^
**General teaching**	59 (52.2%)	33 (54.1%)	22 (51.2%)	
**General non-teaching**	21 (18.6%)	14 (23.0%)	5 (11.6%)	

**Hospital ownership**				
**Public**	89 (78.8%)	46 (74.2%)	36 (83.7%)	0.127^$^
**Private not profit**	10 (8.8%)	5 (8.1%)	5 (11.6%)	
**Private for profit**	14 (12.4%)	11 (17.7%)	2 (4.7%)	

**Hospital beds**				
**Mean (median)**	445 (333)	440 (323)	506 (338)	0.104*

Maturity Index**				
**Mean (SD)**	2.58 (0.69)	2.57 (0.36)	2.61 (0.29)	0.510*
**Median**	2.61	2.59	2.64	
**Min**	1.7	1.7	1.92	
**Max**	3.26	3.26	3.18	

^$^Fisher's Exact Test; * U-Mann-Whitney Test, ** MI scale: 0 (highest maturity) to 4 (lowest maturity); ^◆ ^*n = 105 hospitals*

The distribution of hospitals in the MARQUIS-IASIST study is not statistically different to the distribution of hospitals participating in MARQUIS SPAIN alone, based on the characteristics explored here: type of hospital, ownership, number of beds, MI. It seems that there is an overrepresentation of university hospitals and under-representation of private-for profit hospitals, but the differences in the data set are not significant. However, comparing the data to the hospitals in Spain at large we observe a higher representation of larger and public hospitals in our sample.

For the interpretation of the variable 'maturity index' it should be noted that a value of 1 reflects the best possible value, e.g. a hospital performing high on all 113 items of the score. Likewise, the value of 4 reflects the lowest possible value of a hospital on the items of the index. The score is an aggregate mean score of items in the seven dimensions which are weighted according to the Plan-Do-Study-Check (PDCA) cycle; that is, it is not a simple yes-or-no assessment of compliance with each of the items. In Table 3 we present measures of central tendency and dispersion for the dependent variables. For each variable we indicate mean, standard deviation, minimum and maximum values, and the inter-quartile range.

**Table 3:** Distribution of independent variables: hospital level indicators of quality and patient safety

	Descriptive statistic		
Indicator	Mean (SD)	Min-max	Inter-quartile range
Adjusted hospital mortality index*	1.03 (0.51)	0.55-3.72	0.79-1.03
**Adjusted hospital complications index**	0.94 (0.27)	0.02-1.94	0.84-1.01
**Adjusted hospital readmissions index**	1.02 (0.20)	0.13-1.28	0.93-1.14
Adjusted hospital length of stay index*	1.01 (0.28)	0.75-2.50	0.86-1.04

*Distribution not normal (Kolmogorov-Smirnov test for normality: p < 0.05)

The indicators presented in Table 3 are ratio-based (observed over expected). For example, an adjusted hospital mortality rate of 1 would suggest that mortality is exactly at the level that could be expected for the type of patients treated in the hospital, a rate of 2 would indicate that it is twice as high as expected. These ratios are adjusted for patient and organizational characteristics; however, they might be influenced for example by specific referral, local admission and/or discharge policies that are not captured in the model, and hence do not automatically mean that an individual hospital provides higher or lower quality of care.

The means differ slightly from 1 since the data reflects a sub-sample of the IASIST data that was used to calculate the indicators. We checked the distributions of data and performed tests of normality using the Kolmogorov-Smirnov test. We identified various hospitals with outliers for the four indicators (as reflected by the minimum and maximum values). Since these outliers are not data entry errors but rather reflect real life variations in the indicators we retained them in further analysis. Nevertheless, we adjusted our data analysis strategy considering that high leverage on the predictor variables can have an influential effect on the correlation and regression coefficients.

In the second step, we calculated correlations between hospital MI and hospital level indicators using both traditional correlations coefficients (Pearson or Spearmen, depending on the distribution of the data) and the bootstrapping technique. Using the bootstrapping procedure in *R*, we drew 1000 repeated samples of equal size (with replacement) of our data and plotted the distribution of the newly derived data (see Additional File 2 Annex 2, paragraph 1). Results of the correlation analysis and bootstrapping procedures are presented in Table 4. The conventional correlation analysis using Pearson's or Spearman's coefficients indicate significant associations between MI and the dependent variables of hospital complications and hospital readmissions. The bootstrap analysis slightly modifies the results of the Pearson and Spearman correlation coefficients by better addressing the effects of influential outliers in the samples taken. Adjusted hospital complications remain correlated with the maturity of the hospitals' quality improvement system (MI) while adjusted hospital readmissions are only borderline significant in the bootstrap analysis. According to both correlation and bootstrap analysis, adjusted hospital mortality and adjusted hospital length of stay do not reveal any discernible correlation. To illustrate the values for each of the four independent variables and the predictor variable MI we provide scatter plots.

**Table 4:** Correlation and bootstrap analysis between hospital level indicators and maturity index (MI)

Indicator	Correlation coefficient for MI (p-value)	Bootstrap estimate (p-value)
**Adjusted hospital mortality index**	**-0,155 **(0,320)	**-0,42 **(-0,66; 0,12)

**Adjusted hospital complications index**	**0,327 **(0,032)*	**0,32 **(0,003; 0,65)*

**Adjusted hospital readmissions index**	**0,322 **(0,035)*	**0,31 **(-0,09; 0,58) ^&^

**Adjusted hospital length of stay index**	**0,001 **(0,996)	**-0,39 **(-0,66; 0,27)

* = statistically significant at p < 0.05; ^&^= borderline statistically significant

**Figure 2:**
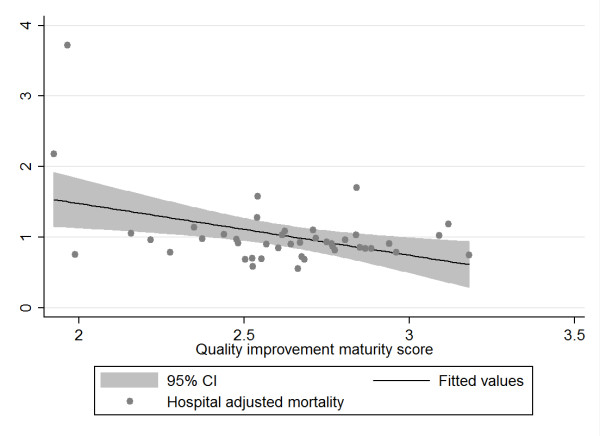
**Scatter plot of hospital adjusted mortality rate and hospital quality improvement system maturity index (MI)**.

Figure 2: Association between hospital quality improvement maturity score and hospital adjusted mortality

**Figure 3:**
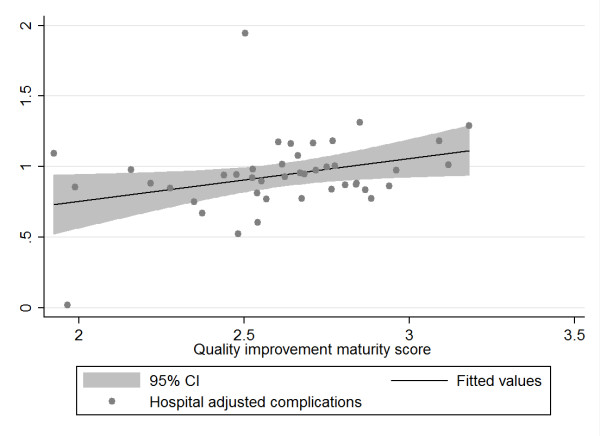
**Scatter plot of hospital adjusted complication rate and hospital quality improvement system maturity index (MI)**.

Figure 3: Association between hospital quality improvement maturity score and hospital adjusted complications

**Figure 4:**
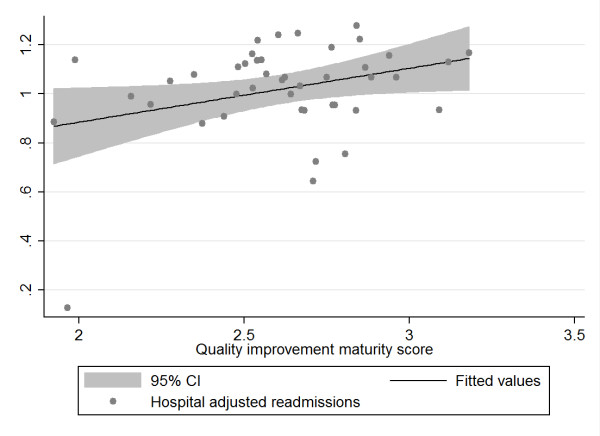
**Scatter plot of hospital adjusted readmission rate and hospital quality improvement system maturity index (MI)**.

Figure 4: Association between hospital quality improvement maturity score and hospital adjusted readmissions

**Figure 5:**
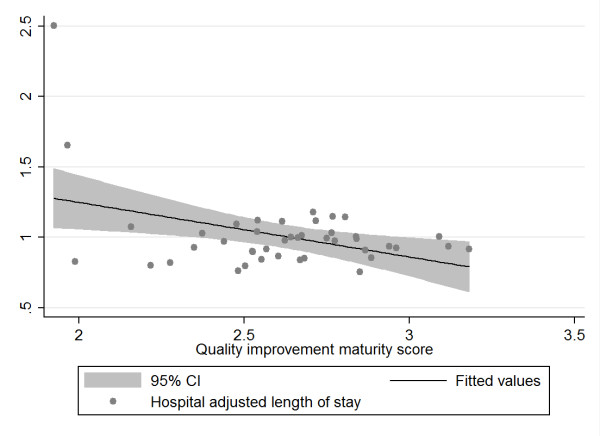
**Scatter plot of hospital adjusted length of stay rate and hospital quality improvement system maturity index (MI)**.

Figure 5: Association between hospital quality improvement maturity score and hospital adjusted length of stay

The scatter plots (with fitted values and 95% confidence intervals) reflect the data from the bootstrap analysis and indicate a negative relationship between adjusted hospital mortality and adjusted hospital length of stay and MI on the one hand and a positive relationship between adjusted hospital complications and readmissions and MI on the other hand. Given that an MI score of 1 reflects the best possible quality improvement system, the data suggest that hospitals with the best MI have the lowest complications and readmissions, but also the highest mortality and length of stay, although this fails to show statistical significance. The plots above displayed various outliers, reflecting different hospitals for each of the variables analyzed.

In a final step, in order to adjust for the effect of potential confounding factors for associations between MI and the dependent variables, we entered the following variables into a multiple regression model: hospital type, ownership, and size. To this end, we created dummy variables for hospital type (university vs non-university), ownership (public vs private) and for size (small, medium, large; based on the distribution of the number of beds in terciles). We used hierarchical variable entry with step 1 comprising the variable MI, and adding the dummy variables for hospital type, ownership and size in step 2. Where indicated, Box-Cox transformations were performed to obtain normality of the dependent variables. Because of the effect that outliers have on the coefficients in the regression analysis when using the ordinary least squares method we applied a regression model that, by reducing the weight of outlying cases, produces robust estimates. Two estimation methods were used (Huber and bi-weighting) and compared to the estimates of the ordinary least square regression model. We then chose the model with the lowest standard error of residuals (the Huber method exhibited the lowest standard error for the model residuals for all indicators except adjusted hospital readmissions, see Additional File 2 Annex 2). Finally, we performed the bootstrapping procedure on the coefficients of these models (Table 5).

**Table 5:** Effect of maturity of the hospital quality improvement system (MI) and hospital structural characteristics on hospital level indicators

Dependent variable	Predictor variables	B	B (95% confidence interval for the Bootstrap estimate)
**Hospital adjusted mortality index^&^**	**Step 1**		
	MI	0.199	0.195 (-0.175; 0.597)
	
	**Step 2**		
	MI	0.195	0.191 (-0.202; 0.643)
	Hospital type	-0.001	-0.001 (-0.596; 0.560)
	*University*	-	-
	*Non University*	-	-
	Hospital size	0	0.001 (-0.338; 0.326)
	*Small*	0.002	0.004 (-0.586; 0.569)
	*Medium*		
	*Large*		

**Hospital adjusted complications index**	**Step 1**		
	MI	0.245	0.240 (-0.006; 0.580)*
	
	**Step 2**		
	MI	0.247	0.242 (0.009; 0.595)**
	Hospital ownership	0.003	0.003 (-0.192; 0.182)
	*Public*	-	-
	*Private*		

**Hospital adjusted readmissions index**	**Step 1**		
	MI	0.077	0.113 (-0.092; 0.283)*
	
	**Step 2**		
	MI	0.075	0.120 (-0.111, 0.299)
	Hospital size	-	-
	*Small*	0	0.000 (-0.230; 0.268)
	*Medium*	-0.002	-0.006 (-0.230; 0.268)
	*Large*		

**Hospital adjusted length of stay index^&&^**	**Step 1**		
	MI	0.078	0.069 (-0.320; 0.574)

**^&^**Box-Cox transformation: 1/hospital adjusted mortality

**^&&^**Box-Cox transformation: (1/hospital adjusted length of stay^2^

**significant

*borderline significant

**Figure 6:**
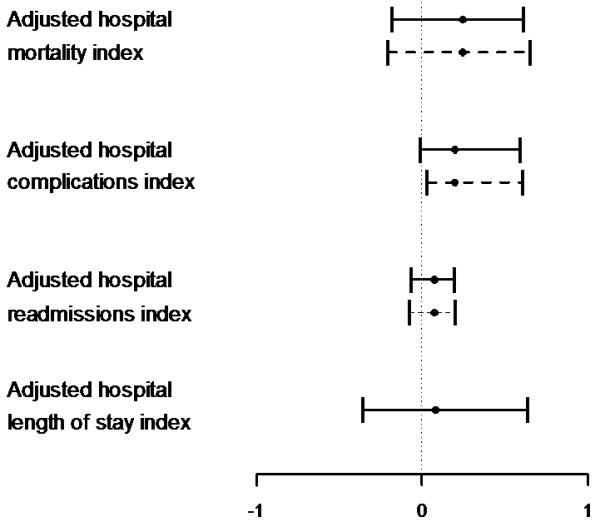
Effect of maturity index (95% CI) on hospital-level indicators*. *Straight line denotes B value and 95% confidence interval for the Bootstrap estimate for the unadjusted analysis; the dotted line provides this information for the adjusted analysis.

For adjusted hospital mortality, the robust regression analysis confirms previous bi-variate analysis. The confidence intervals for the B values show no significant effect of MI, even after adjusting for type and size of hospital. The model for hospital adjusted complications shows a borderline significant effect of MI which becomes significant after adjusting for hospital ownership. In the case of adjusted hospital readmissions, the borderline significance for MI is further diminished after accounting for the factor hospital size. In the final model for hospital adjusted length of stay, neither MI nor any of the covariates proved to be significant (Figure 6).

## Discussion

This is the first analysis linking the 'maturity' of the hospitals' quality improvement system score (MI) to quality and patient safety outcomes in 43 Spanish hospitals. The data suggests that adjusted hospital complications are associated with the development of the hospitals' quality improvement systems: hospitals with more mature quality improvement systems present lower complication rates. Similar results, although borderline significant and partly confounded by hospital size can be observed for adjusted hospital readmissions. Perhaps contrary to common expectations we found the associations between MI and the dependent variables of mortality to be negative, meaning that hospitals with a more mature quality improvement system had higher mortality rates than other hospitals. Even though this association was not significant it raises issues about the use of the MI to identify hospitals with high mortality. Adjusted hospital length of stay was not significantly associated with MI, probably because of insufficient variability in the data set with regard to this variable.

### How do we explain the associations?

The link between MI and hospital adjusted complications was corroborated systematically in our analysis and was irrespective of level of analysis (bi-variate correlation vs. multivariate regression) or estimation method (ordinary least square regression, Huber estimation or bi-weight estimation). Hospital complication indicators have been computed based on administrative data for more than 15 years and risk-adjustments have matured substantially over this period [22]. The associations detected between MI and hospital adjusted complications are plausible given the number of items of the MI directed at establishing monitoring and reporting systems, implementing protocols to improve clinical effectiveness and patient safety and designating responsibilities for a number of key patient safety issues (infections, blood transfusion, and prevention of decubitus ulcer, etc). This finding is important considering that hospital complications are at best a cause of irritation and frustration to patients and more likely put their life at risk, in addition to leading to increased hospital expenditure [23].

The link between MI and readmission is much weaker in our data and at best borderline significant. It seems that it is more difficult to establish a relation between the few items related to discharge planning in the MI and the readmission indicator. Difficulties in discriminating between hospital readmission rates based on their discharge procedures were also demonstrated by recent research in the USA [24], even though such activities have proven to be effective in clinical trials [25]. Generalization of the research that was performed on this topic in the USA to the situation in Spain is limited, however, considering the comparatively well-developed primary care system in Spain.

Mortality rates have for a long-time been discussed as to whether they are a valid measure of hospital performance. Since the introduction of the standardized hospital mortality rates and subsequent refinements of the methods for its calculation [26,27], adjusted hospital mortality ratios have been considered by many as an inclusive flag of hospital quality that should require further investigation. From a research perspective, it is not too surprising that adjusted hospital mortality is not related to MI in our study, given the methodological challenges that limit its use, mainly driven by the low signal to noise ratio and subsequent problems of risk adjustment, such as the case-mix adjustment fallacy or constant risk fallacy [28]. In fact, quality of care only accounts for small variations in mortality and the quality improvement systems are a distal factor modifying quality of care [29]. In addition, differences in quality of care within hospitals might be greater than between hospitals and may attenuate any possible association between the quality improvement systems and quality and patient safety outcomes in concrete diagnoses.

For adjusted hospital length of stay we did not detect any relationship with MI. It is possible that the variability of the data is too limited to establish any associations. On the other hand, the link between maturity of the quality improvement systems and length of stay is less explored and the direction of the associations, if it existed, is unclear. Length of stay, while a common indicator in assessing hospital performance [30], is prone to a number of biases that are very difficult to disentangle, mainly relating to the local organization of the hospital in the care network which makes it difficult to interpret the indicator.

### Limitations of the study

The research presented here has some limitations which are discussed below. The sample size of this study is limited. From the initial 51 hospitals that participated in both the MARQuIS study and IASIST, we obtained consent from only 43 hospitals to match the data from both. We observed some outliers which we retained in the analysis; firstly because there was no conclusive reason to exclude these hospitals since the data do not appear to be erroneous, and secondly, in order not to reduce the sample size further. To account for the effect of the outliers and to obtain more robust estimates for the correlation coefficients we applied the bootstrapping procedure to resample hospitals from our database. In addition, we used robust estimation methods for the regression analysis to ensure that the coefficients are not inflated by the outliers or observations with high leverage. Although we did adjust the multiple regression analysis for a number of potential confounding factors such as type, ownership and size of the hospital, we were not able to adjust for additional factors known to be related to hospital outcomes, like nurse-patient ratios or organizational culture [31-33]. Moreover, while the independent variables have undergone extensive validation, further research may be necessary to increase the sensitivity of the maturity index to detect differences in quality and patient safety outcomes at hospital level. Finally, the generalisability of the findings to Spanish hospitals at large, including the high number of specialized private-for-profit clinics, is limited considering the overrepresentation of larger and public hospitals in our sample.

### Implications for research

Despite the limitations outlined above, we believe that the results presented here raise some important questions for further research on exploring the impact of quality improvement systems on patient level outcomes. This is particularly so since our results are in line with previous research that demonstrates some effects of quality improvement systems on hospital compliance with quality criteria or on patient level outcomes, even though this effect is not always systematic [34] nor its direction conclusive [8]. Most of the problems in linking the effect of quality improvement systems on outcomes, in summary, are due to the fact that many components of a quality improvement system are only a distal factor to quality and patient safety outcomes and therefore demonstration of its impact is prone to a variety of methodological issues.

In order to advance the research on quality improvement we propose the following. Firstly, research should target the development of a valid, reliable and feasible instrument to assess the quality improvement system. The maturity index used in this study or comparable measures being developed elsewhere [7,35-37] provide a good starting point, but further validations are required, potentially incorporating item-reduction strategies [38,39]. Ideally, assessment of the quality improvement system in itself would become a routine indicator collected by hospitals alongside the range of performance indicators being periodically collected. Secondly, given that quality improvement activities take place throughout the hospital, the relationship between hospital-wide and departmental-specific quality improvement activities should be explored. Considering that departmental level activities are more proximal to outcomes, it might be possible to detect stronger associations and prevent attenuation of within-hospital variations in quality outcomes [40]. Thirdly, assessments of outcomes should be accompanied by assessments of clinical processes against evidence-based standards for effective, safe and patient-centred care. Attribution of quality improvement activity to outcomes might be supported by further breaking down assessment of processes into generic management processes, targeted management processes and clinical processes [41]. Fourthly, quality improvement research may need to better address the factors potentially confounding the associations explored. Since the feasibility of assessing all necessary information (such as hospital structural characteristics, implementation of clinical information systems, organizational culture, patient safety culture, nurse-to-patient ratios, the market environment etc.) in a large sample of hospitals is very limited, quality improvement research should explore recent advances in epidemiological modeling to assess the effect of unmeasured confounders [42,43]. Finally, research on the effectiveness of quality improvement systems should explore the key aspects and latent dimensions of quality improvement that are mostly related with quality and patient safety outcomes. The research agenda to link structure, process and outcomes in health care, as introduced by Donabedian over 40 years ago, thus continues. Some of these challenges are further pursued in the EU FP7 funded research collaborative 'Deepening our Understanding of Quality Improvement in Europe [DUQuE]' [44].

## Conclusion

In our analysis of 43 Spanish hospitals we found the maturity of the hospitals' quality improvement to be consistently associated with adjusted hospital complications and to some extent with adjusted hospital readmissions, while adjusted hospital mortality and adjusted hospital length of stay were not found to be associated. From this data it seems that hospitals with more mature quality improvement system have lower rates of complications. A number of methodological and logistic hurdles remain to link hospital quality improvement systems to outcomes. Further research should address these and the quality improvement agenda should continue to include research on the effectiveness of quality improvement to demonstrate and justify its value.

## Competing interests

The authors declare that they have no competing interests.

## Authors' contributions

OG and RS conceived of the study. MS and MC collaborated in the design of the study. OG, NM and AT performed the statistical analysis. OG drafted the manuscript. All authors critically read, revised and approved the final manuscript.

## Pre-publication history

The pre-publication history for this paper can be accessed here:

http://www.biomedcentral.com/1472-6963/11/344/prepub

## Supplementary Material

Additional file 1 **Definition of hospital adjusted complications**. List of complications included in the calculation of the indicator 'hospital adjusted complications'. Click here for file

Additional file 2 **Statistical specifications**. Statistical specification and links to the R syntax for the bootstrapping procedure, robust regression procedure and Box-Cox transformation procedure. Click here for file
