# Supplementary material for: Is the maturity of hospitals' quality improvement systems associated with measures of quality and patient safety?
Source: BMC Health Serv Res. 2011 Dec 20;11:344. doi: 10.1186/1472-6963-11-344 (PMC3267703; doi:10.1186/1472-6963-11-344)
Supplement: Additional file 1 — Definition of hospital adjusted complications. List of complications included in the calculation of the indicator 'hospital adjusted complications'. [file 1472-6963-11-344-S1.DOC]

# Additional file 1

# Annex 1: List of complications included in the calculation of the indicator ‘hospital adjusted complications’

**Complications**

- Risk adjusted complications
- Postoperative complications related to the urinary tract
- Postoperative pulmonary complications
- Postoperative gastro-intestinal hemorrhage or ulceration after non-gastrointestinal surgery
- Decubitus ulcer
- Postoperative septicemia, abscess or wound infection
- Aspiration pneumonia
- Postoperative cardiac abnormalities (except after acute myocardial infarction)
- Mechanical complication due to a device, implant or graft (except after organ transplantation)
- Miscellaneous surgical complications
- Shock after or during operation due to anesthesia
- Postoperative complications related to the central or peripheral nervous system
- Postoperative acute myocardial infarction
- Perforation or laceration related to a procedure
- Postoperative physiological or metabolic disorder
- Postoperative stupor or coma
- Postoperative pneumonia
- Complications related to anesthesia agent or other depressors of the central nervous system
- Venous thrombosis or pulmonary embolism
- Hemorrhage or hematoma after procedure
- Fall of hip fracture within the hospital
- Complication after procedure of other organ systems
- Medication related complications
- Complications related to organ transplantation
- Reopening of a surgical wound

**Sentinel events**

- Gas gangrene
- Abscess of the central nervous system
- Anoxious brain lesion
- Foreign body accidentally left during procedure
- Reaction of incompatibility to antigen
- Reaction of incompatibility of Rhesus factor
- Gas embolism that complicates medical attention
- Acute reaction to a foreign substance accidentally left during the procedure
