# Supplementary material for: Is the maturity of hospitals' quality improvement systems associated with measures of quality and patient safety?
Source: BMC Health Serv Res. 2011 Dec 20;11:344. doi: 10.1186/1472-6963-11-344 (PMC3267703; doi:10.1186/1472-6963-11-344)
Supplement: Additional file 2 — Statistical specifications. Statistical specification and links to the R syntax for the bootstrapping procedure, robust regression procedure and Box-Cox transformation procedure. [file 1472-6963-11-344-S2.DOC]

# Additional file 2

# Annex 2: Statistical Annex

This statistical annex provides additional information on the statistical values for the dependent variables (point 1), the bootstrapping procedure (point 2), the robust regression analysis (point 3) and Box-Cox transformation (point 4) in the R software package.

1. **Statistical values for the dependent variables**

**Table A:** Statistical values for the dependent variables

| **Indicator** | **Sensitivity** | **Specificity** | **Receiver operating characteristic (ROC)** | **R^2^** |
| --- | --- | --- | --- | --- |
| **Hospital adjusted mortality index** | 0.90 | 0.83 | 0.94 | NA |
| **Hospital adjusted complications index** | 0.72 | 0.86 | 0.86 | NA |
| **Hospital adjusted readmissions index** | 0.71 | 0.69 | 0.76 | NA |
| **Hospital adjusted length of stay index** | NA | NA | NA | 47.6% |

1. **Bootstrapping procedure**

Bootstrapping is a re-sampling method that allows estimating the precision of a particular statistic by drawing repeated samples from a defined population. We performed this procedure drawing repeated samples of 1000 (with replacement) using the boot command in the R statistical package [1].

To illustrate the bootstrapping procedure, Figure 1 shows the distribution of the correlation coefficient between the dependent variable ‘hospital adjusted complications’ and the independent variable ‘MI’. The horizontal axis represents the value of the correlation coefficient and the vertical axis represents its relative frequency*10 (as %). Considering this distribution to reflect a continuous, random variable, the interpretation should focus on the intervals for the values and not the individual values. The bars of the histogram, for example in case of the highest bar marked below, indicates that the estimated correlation coefficient takes values between 0.3 and 0.4 in more than 20% of the samples taken. The percentiles 2.5 and 97.5 of the distribution reflect the 95% confidence interval for the correlation coefficient, which in this case have the value 0.003 to 0.65 around a mean of .327.

Figure a): Bootstrap distribution of the correlation coefficients for ‘hospital adjusted complications’ and MI (n=1000)


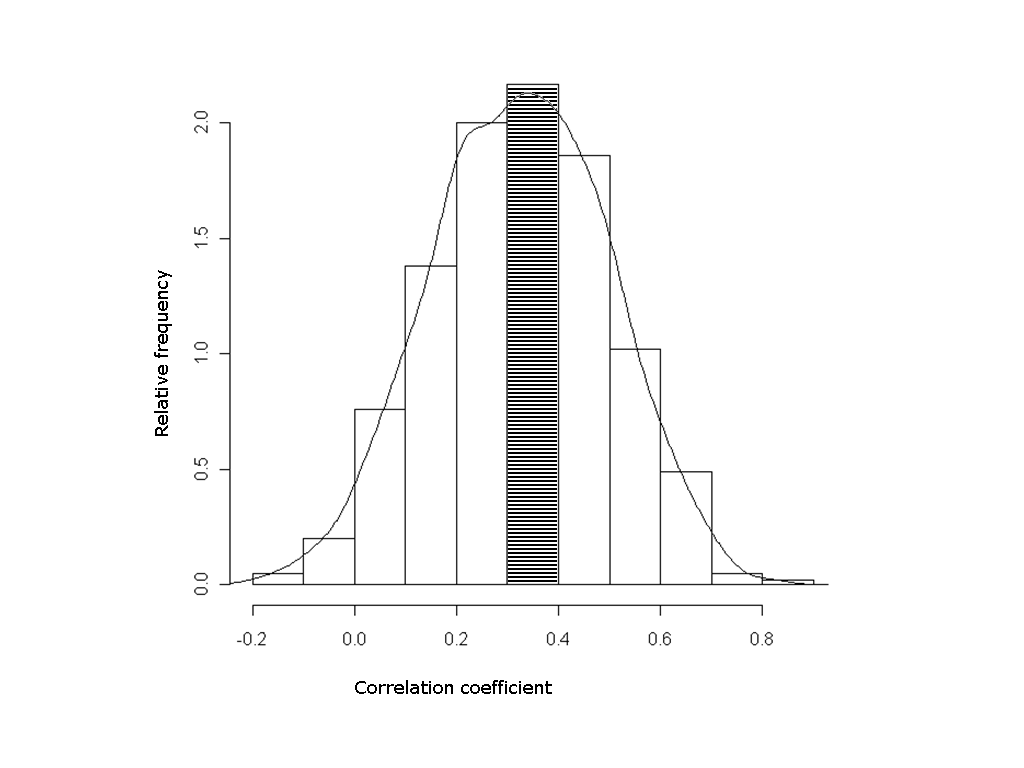


1. **Robust regression**

Robust regression is a method that is used to estimate the parameters of a model when the data present outliers that could have a strong influence on the estimates. Various methods of robust regression exist but the most common ones are the Huber and Bi-weighting estimation methods. Both methods aim at minimizing the variance of the residuals by assigning specific weights to each observation. The differences between both methods lie in the assignation of weights.

Table B shows the weighting function for the ordinary least square estimation, Huber and bi-weighting method. The estimations for the regression models were performed using the *rlm* function in the R statistical software package [2]. The final model was selected based on the intention to minimize the estimates of the standard error of residuals. All estimates are presented in Table C.

**Table B**: Weighting function for the ordinary least square estimation, Huber and bi-weighting method

| **Method** | **Weight Function** |
| --- | --- |
| Least-Squares | 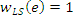 |
| Huber | 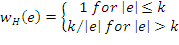 , where 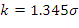 |
| Biweight | 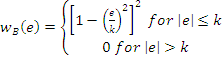 , where 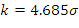 |

**Table C:** Standard error of the residuals for each of the regression estimation methods (ordinary least square (OLS), Huber weighting and bi-weighting) and each independent variable

|  |  | **Standard error of the residuals** | | |
| --- | --- | --- | --- | --- |
| **Variable** |  | **OLS** | **Huber** | **Biweight** |
| ***Mortality*** | ***Crude*** | 0.29 | 0.18** | 0.21 |
|  | ***Adjusted*** | 0.27 | 0.26 | 0.25 |
| ***Complications*** | ***Crude*** | 0.26 | 0.18** | 0.16 |
|  | ***Adjusted*** | 0.25 | 0.18 | NA |
| ***Readmissions*** | ***Crude*** | 0.19 | 0.15 | 0.14** |
|  | ***Adjusted*** | 0.18 | 0.26 | 0.11 |
| ***Length of stay*** | ***Crude*** | 0.32 | 0.24** | 0.29 |
|  | ***Adjusted*** | NA | NA | NA |

**Model selected

1. **Box-Cox transformation**

The **Box-Cox transformation** is a function of a random variable X that is computed to obtain normality or homocedasticity (constant variance) of a variable. Its expression is:


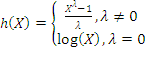
.

The constant
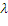
was estimated using maximum verisimilitude in the function *boxcox* of the R statistical software package [3].

# References

1. R statistical package Bootstrapping procedure: <http://127.0.0.1:21872/library/boot/html/boot.html>
2. R statistical package Robust Regression Weighting Function: <http://127.0.0.1:21980/library/MASS/html/rlm.html>
3. R statistical package BoxCox function: <http://127.0.0.1:21980/library/MASS/html/boxcox.html>
